# Comparison of Segmental Thoracic and Lumbar Subarachnoid Block in Preeclamptic Patients Undergoing Cesarean Section: An Open-Label Randomized Trial

**DOI:** 10.7759/cureus.89190

**Published:** 2025-08-01

**Authors:** Anshul Jain, Shivali Pandey, Fahad Suhail, Sippy Agarwal, Divya Jain, Charu Thakur

**Affiliations:** 1 Anesthesiology, Maharani Laxmi Bai Medical College, Jhansi, IND; 2 Obstetrics and Gynecology, Maharani Laxmi Bai Medical College, Jhansi, IND

**Keywords:** category 2 cesarean section, hemodynamic stability, preeclampsia-eclampsia, thoracic segmental spinal anesthesia, cesarean section

## Abstract

Background

Preeclampsia is a serious hypertensive disorder of pregnancy characterized by multi-organ dysfunction and often necessitates cesarean delivery. While spinal anesthesia is preferred for cesarean sections, lumbar subarachnoid block may result in significant hypotension due to extensive sympathetic blockade. Thoracic spinal anesthesia has emerged as a potential alternative with better hemodynamic control.

Objective

To compare the efficacy, safety, and hemodynamic effects of thoracic versus lumbar subarachnoid block in preeclamptic patients undergoing cesarean delivery.

Methods

In this prospective, open-label, randomized controlled trial, 160 preeclamptic patients scheduled for urgent cesarean sections were allocated to receive either thoracic spinal anesthesia (1.5 mL 0.5% bupivacaine heavy + 25 μg fentanyl) or lumbar spinal anesthesia (2 mL 0.5% bupivacaine heavy + 25 μg fentanyl). The primary outcome was hemodynamic stability; secondary outcomes included surgical anesthesia success, block onset and duration, adverse events, neonatal outcomes, and patient satisfaction. Data were analyzed using appropriate statistical tests, with p<0.05 considered significant.

Results

Hemodynamic stability was superior in the thoracic group, with a lower incidence of hypotension (11.68% vs. 26.31%, p=0.038) and significantly reduced mean vasopressor (ephedrine) requirement (8.57 ± 1.32 mg vs. 14.41 ± 2.62 mg, p < 0.001). Surgical anesthesia success rates were similar in both groups (thoracic: 96.67%, lumbar: 95%, p=1.0). Thoracic spinal anesthesia was associated with shorter sensory (132.61 ± 13.81 vs. 148.48 ± 14.52 min, p=0.001) and motor block durations (102.23 ± 11.13 vs. 124.01 ± 11.01 min, p=0.001), less shivering and nausea, and greater patient satisfaction. Neonatal Apgar scores and 6-week outcomes were comparable between groups (p>0.05).

Conclusion

Thoracic spinal anesthesia provides superior hemodynamic stability and better patient comfort with comparable surgical efficacy and neonatal safety, making it a viable option for cesarean delivery in preeclamptic patients when administered by experienced clinicians.

## Introduction

Preeclampsia is a major pregnancy complication, characterized by hypertension (systolic ≥140 mmHg and/or diastolic ≥90 mmHg) and proteinuria (>300 mg/24 hours) after 20 weeks of gestation in previously normotensive women [1]. It is the third most common direct cause of maternal mortality, contributing to 9-26% of maternal deaths globally [2].

Elective cesarean section is the most frequent delivery method in preeclamptic women, with spinal anesthesia as the preferred technique [3]. However, emergency cesarean sections are often required, and anesthetic choice depends on urgency. The Lucas classification (2000) categorizes cesarean sections into four levels of urgency: Category 1 (immediate threat to mother or fetus), Category 2 (maternal/fetal compromise without immediate life threat), Category 3 (early delivery needed without compromise), and Category 4 (elective) [4]. General anesthesia with rapid sequence intubation is preferred for Category 1, while for Categories 2 and 3, choices vary. Some centres opt for general anesthesia to prevent exaggerated hemodynamic responses, whereas others prefer regional anesthesia for advantages such as reduced bleeding, dense analgesia, minimal fetal drug exposure, and improved maternal experience [5].

Subarachnoid blocks performed via the conventional lumbar route, however may be associated with significant hypotension due to extensive sympathetic blockade. This is particularly concerning in preeclamptic parturients, where hemodynamic stability is critical. Segmental thoracic spinal anesthesia, performed at mid- to lower thoracic levels, has been successfully used in a range of surgeries, including abdominal, orthopedic, and breast procedures [6-8]. Recent case series and feasibility reports suggest its safe application in obstetric anesthesia, particularly when lumbar access is technically challenging or when better segmental control is desired.

Compared to the lumbar approach, thoracic spinal anesthesia allows for lower anesthetic doses and potentially more restricted sympathetic blockade. Previous studies have shown that segmental thoracic blocks with low-dose bupivacaine result in limited sympathetic spread and improved hemodynamic profiles in high-risk patients [6,9]. These advantages may be particularly beneficial in preeclamptic patients who are already at risk for exaggerated cardiovascular responses.

We hypothesized that segmental thoracic spinal anesthesia, using a lower volume of local anesthetic, would provide superior hemodynamic stability with comparable block efficacy and safety compared to lumbar spinal anesthesia in preeclamptic parturients undergoing cesarean section. To test this hypothesis, we conducted a prospective, open-label, randomized controlled trial. This study investigates whether thoracic spinal anesthesia provides superior hemodynamic stability compared to lumbar spinal anesthesia in preeclamptic patients undergoing Category II and III cesarean sections.

The aim of the study was to compare the efficacy and safety of thoracic versus lumbar spinal anesthesia in preeclamptic patients undergoing cesarean section, with a focus on hemodynamic stability, block characteristics, maternal, and fetal outcomes. The primary objective was to compare hemodynamic stability between thoracic and lumbar subarachnoid blocks. The secondary objectives included evaluating sensory and motor blockade onset and duration, postoperative analgesia, neonatal outcomes, intraoperative adverse effects, and maternal satisfaction.

## Materials and methods

Study design and subjects

The current study was designed as a prospective, open-label randomized trial involving patients with preeclampsia undergoing cesarean delivery. The study was conducted between October 2023 and July 2024. The study protocol and procedure were approved by the Institute Ethics Committee, Maharani Laxmi Bai Medical College (Approval No. 1090/IEC/1/2022-2023) and adhered to the principles outlined in the Declaration of Helsinki (2013). Written informed consent was obtained from all participants prior to enrolment. The study was prospectively registered with the Clinical Trials Registry-India (CTRI/2023/10/058993, dated 20/10/2023) and followed the CONSORT checklist for the preparation of this manuscript.

Participants were prebooked patients with preeclampsia who met the case-defining criteria (Table 1) established by the American College of Obstetricians and Gynecologists [1]. The study was conducted in the dedicated obstetric operation theatre of a tertiary care teaching hospital, equipped with advanced monitoring systems, anesthesia workstations, and resuscitation facilities.

**Table 1:** Case-defining diagnostic thresholds for preeclamsia used in the study

Criteria	Diagnostic threshold
Blood pressuren (BP)	Systolic BP ≥ 140 mmHg or diastolic BP ≥ 90 mmHg on two occasions at least 4 hours apart after 20 weeks of gestation
	Systolic BP ≥ 160 mmHg or diastolic BP ≥ 110 mmHg. Severe hypertension can be confirmed within minutes for treatment
Plus A or B	
A. Proteinuria (any one)	≥ 300 mg per 24-hour urine collection (or equivalent from a timed collection)
	Protein/creatinine ratio ≥ 0.3 mg/dL
	Dipstick reading of 2+ (only if other quantitative methods are unavailable)
B. Non-proteinuric criteria (in the absence of proteinuria, new-onset hypertension with any one)	Thrombocytopenia: Platelet count < 100,000 × 10⁹/L
	Renal insufficiency: Serum creatinine > 1.1 mg/dL or doubling of serum creatinine without other renal disease
	Impaired liver function: Elevated liver transaminase concentrations to twice the normal level
	Pulmonary edema
	Neurological symptoms (new-onset headache unresponsive to medication or visual disturbances not explained by other causes)

Inclusion and exclusion criteria

Patients aged between 20 and 40 years, diagnosed with preeclampsia, and classified as American Society of Anesthesiologists (ASA) Physical Status II or III were included in the study.

Patients were excluded if they required emergent cesarean section (Category 1 indication), had a height less than 150 cm or greater than 180 cm, or weighed less than 60 kg or more than 90 kg. Additional exclusion criteria included coagulopathy, defined as an international normalized ratio (INR) >1.5 or platelet count <50,000/mm³; coexisting cardiac disease or poorly controlled diabetes mellitus (HbA1c >9%); pre-existing neurological or psychiatric illness; local infection or inflammation at the intended puncture site; and refusal to undergo subarachnoid block.

Sample size

The sample size was calculated to detect a 20% difference in the incidence of hypotension between thoracic and lumbar spinal anesthesia groups (20% vs. 40%), based on previous literature in similar high-risk populations [10]. Assuming a two-sided alpha level of 0.05 and a power of 90%, the required sample size was 64 patients per group. The formula used for comparing two proportions was:

$n = \frac{(Z_{\alpha/2} + Z_{\beta})^2 \times \left[ P_1(1 - P_1) + P_2(1 - P_2) \right]}{(P_1 - P_2)^2}$

where Z_α/2_=1.96 for 95% confidence, Z_β​_=1.28 for 90% power, P_1​_=0.40 (expected hypotension in lumbar group), and P_2_​=0.20 (expected hypotension in thoracic group). To accommodate for potential dropouts, technical failures, or protocol deviations, the final sample size was increased to 80 patients per group, resulting in a total sample of 160 participants.

Intervention

All participants meeting the study criteria underwent a point-of-care assessment for coagulopathy, including a complete blood count (CBC) and prothrombin time/international normalized ratio (PT/INR). Eligible participants were randomized into two groups using computer-generated randomization.

Group I received a thoracic subarachnoid block with 1.5 mL of 0.5% bupivacaine heavy combined with 25 μg fentanyl administered at the T8-T9 or T9-T10 interspace. Group II received lumbar subarachnoid block with 2 mL of 0.5% bupivacaine heavy combined with 25 μg fentanyl administered at the L3-L4 or L4-L5 interspace.

In both groups, the block was performed by a senior anesthesiologist with experience in over 50 successful thoracic and lumbar subarachnoid procedures. An 18G intravenous cannula was placed in the non-dominant hand, and co-loading with Ringer’s lactate (10 mL/kg) was initiated immediately after intrathecal injection. Standard premedication (25 mg ranitidine, 1 mg granisetron) was given intravenously for reflux prevention and PONV (postoperative nausea and vomiting) prophylaxis. Labetalol (20 mg IV) was administered at 20-minute intervals for patients with a diastolic BP >110 mmHg to lower it below 100 mmHg.

After applying standard noninvasive monitoring (non-invasive blood pressure (NIBP), heart rate (HR), oxygen saturation (SpO₂), temperature, ECG), the puncture site was disinfected with alcoholic chlorhexidine and infiltrated with 1% lidocaine. The block was performed in the sitting position using a 25G BD Quincke spinal needle (Becton, Dickinson and Company, Franklin Lakes, NJ, USA) under aseptic conditions.

Sensory block was assessed using loss of sensation to a 25G blunt needle, starting at the umbilicus (T10) and progressing cephalad to the xiphisternum (T6), beginning at four minutes and repeated every two minutes until 10 minutes. A successful block was defined as achieving a bilateral sensory level at T6 (xiphisternum), after which the incision was allowed. Motor block was assessed bilaterally using the modified Bromage scale (0-3): The scale was defined as follows: 0 - no motor block, 1 - inability to raise extended legs, 2 - inability to flex the knees but able to move the feet, and 3 - inability to flex the ankle joints. Inadequate block height was either supplemented or converted to general anesthesia. Additional analgesia (if needed) to maintain a Visual Analog Scale (VAS) score of <2 was provided using 50 μg of fentanyl. Throughout the procedure, HR, respiratory rate, SpO₂, and ECG were continuously monitored, and BP was recorded every three to five minutes, continuing postoperatively. Hypotension was treated with ephedrine (5 mg IV). After delivery, 2 mg midazolam and 50 mg tramadol IV were administered for analgesia and sedation.

A transversus abdominis plane (TAP) block under ultrasound guidance was performed in both groups using 15 mL of 0.125% bupivacaine on each side for postoperative analgesia. Patients were observed in the post-anesthesia care unit (PACU; 2-4 hours) and followed up on postoperative days 1, 7, and 15 (via phone) for transient neurological symptoms or back pain. 

Outcome measures

The primary outcome was hemodynamic stability, assessed by the incidence of hypotension (defined as >20% fall in systolic blood pressure from baseline), bradycardia (heart rate <60 bpm), total vasopressor requirement (measured as the cumulative dose of ephedrine administered), and episodes of severe hypotension (>30% fall in systolic blood pressure).

Secondary outcomes evaluated the surgical anesthesia success, defined as achieving a sensory block from the xiphisternum to the pubic symphysis, permitting cesarean delivery without the need for additional intraoperative analgesia or conversion to general anesthesia. This was quantified by assessing sensory and motor block onset and duration, along with the intensity of motor block (evaluated using the Modified Bromage Scale). Intraoperative adverse effects: nausea, vomiting, shivering, pruritus, dyspnea were also evaluated along with neonatal outcomes using Apgar scores at 1 and 5 minutes, neonatal intensive care unit (NICU) admission, need for resuscitation or intubation. Patient satisfaction was assessed at 24 hours postoperatively using a 5-point Likert scale (Very satisfied, Satisfied, Neither satisfied nor dissatisfied, Dissatisfied, Very dissatisfied) [11]. Surgeon satisfaction with the anesthetic technique (5-point Likert scale) and postoperative complications, viz., post-dural puncture headache (PDPH), transient neurological symptoms (TNS), and permanent nerve injury.

Statistical methods

Continuous variables were summarized as means ± standard deviations (SD), medians, ranges (minimum to maximum), and 95% confidence intervals (CIs). Categorical variables were described as frequencies and proportions. All statistical analyses were performed using IBM SPSS Statistics for Windows, version 28 (IBM Corp., Armonk, NY, USA). A p-value of <0.05 was considered statistically significant.

## Results

**Figure 1:**
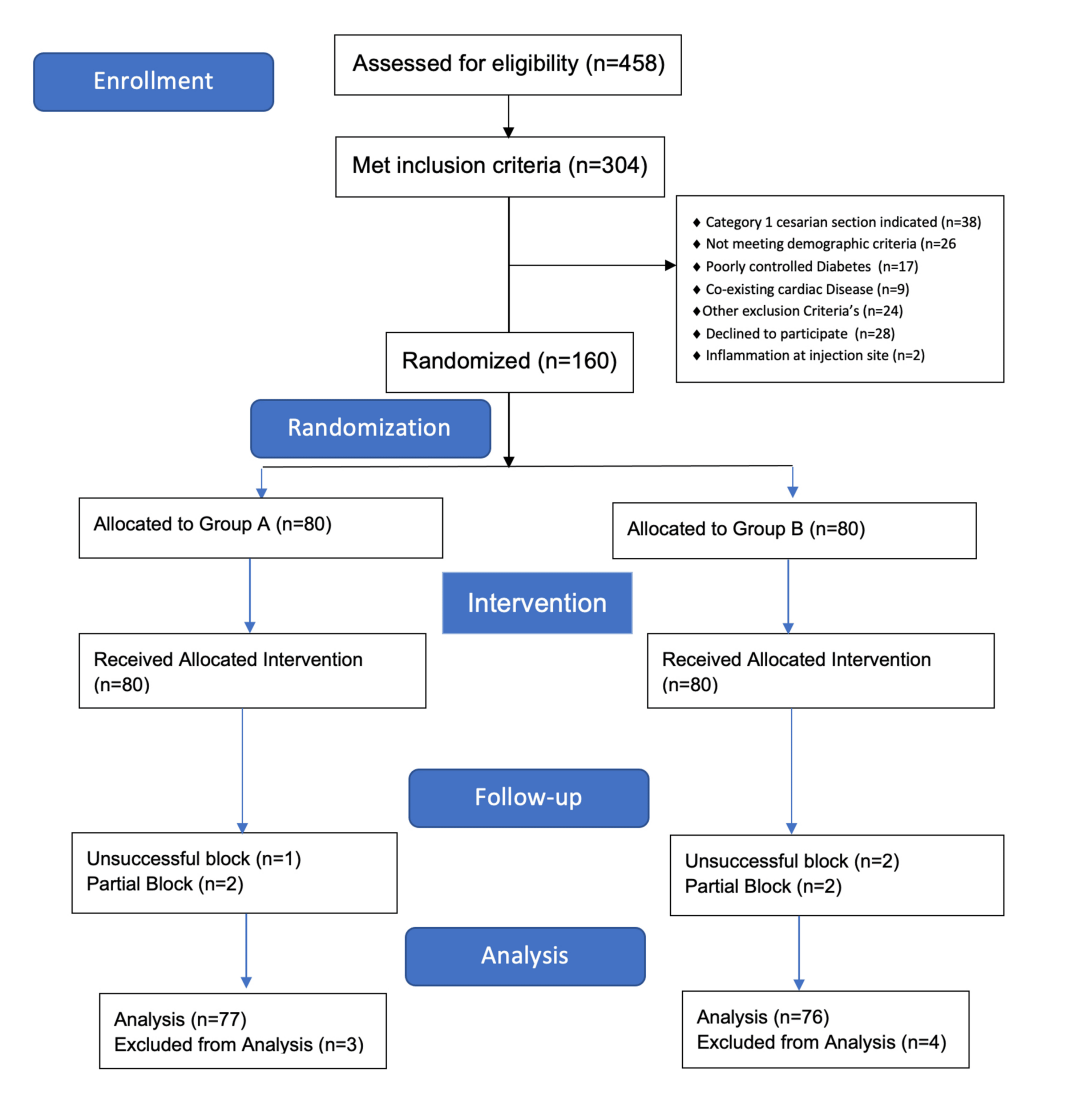
Consolidated Standards of Reporting Trials (CONSORT) diagram

Recruitment for the study occurred between October 2023 and July 2024. During this period, 458 preeclamptic patients scheduled for cesarean section were screened for eligibility. Of these, 304 met the inclusion criteria, while 154 were excluded based on predefined exclusion criteria. Recruitment was stopped once the target sample size was achieved, with the last patient recruited on July 8, 2024. A total of 160 patients were randomized into two groups, as illustrated in the CONSORT (Consolidated Standards of Reporting Trials) flow diagram (Figure 1).

The demographic and baseline characteristics of the participants are summarized in Table 2. The mean age of patients in Group I was 31.03 ± 4.80 years, compared to 29.66 ± 4.71 years in Group II, with no statistically significant difference between the groups (p = 0.07). Similarly, no statistically significant differences were observed in other baseline parameters, including ASA status and category of cesarean section, between the two groups (Table 2).

**Table 2:** Baseline demographic and clinical characteristics of study groups ASA: American Society of Anesthesiologists Data presented as n (%) or Mean ± SD; p < 0.05 was considered significant

Parameters	Group I (N = 80)	Group II (N = 80)	P-value
Age (in years)	31.03 ± 4.80	29.66 ± 4.71	0.070
Weight (in kg)	72.23 ± 6.13	74.01 ± 7.01	0.089
Height (in cm)	158.38 ± 4.92	159.84 ± 5.23	0.07
Category of cesarean section			
Category II (%)	26 (32.5%)	31 (38.75%)	0.40
Category III (%)	54 (67.5%)	49 (61.25%)	0.40
ASA status			
II (%)	42 (52.5%)	47 (58.75%)	0.42
III (%)	38 (47.5%)	33 (41.25%)	0.42
Preeclampsia grade			
Mild	52 (65%)	49 (61.25%)	0.62
Severe	28 (35%)	31 (38.75%	0.62
Baseline vital parameters			
Heart rate (bpm)	91.13 ± 6.93	88.87 ± 8.12	0.06
Systolic blood pressure (mm of Hg)	164.28 ± 14.56	167.81 ± 13.30	0.11
Diastolic blood pressure (mm of Hg)	99.52 ± 8.91	101.83 ± 8.23	0.09

The block was successful in 77 out of 80 patients (96.26%) in Group I and 76 out of 80 patients (95%) in Group II. The difference in block success rates between the two groups was not statistically significant (p = 1.0). The block was established in the majority of patients within 6 minutes (70 in Group I and 65 in Group II), with no statistically significant difference in block achievement time.

The primary outcome, hemodynamic stability, was analyzed in all patients in whom the block was successful (77 in Group I and 76 in Group II). The incidence of hypotension was significantly lower in the thoracic spinal group (11.68%; n=9) compared to the lumbar group (26.31%; n=20) (p = 0.038) (Table 3).

**Table 3:** Comparison of intraoperative and postoperative side effects across groups PDPH: post-dural puncture headache; TNS: transient neurological symptoms Data presented as n (%) or Mean ± SD; #: statistically significant:, p < 0.05 was considered significant

Side-effects	Group I (N=77)	Group II (N = 76)	P value
Intraoperative: number of patients, (%)			
Bradycardia	8 (10.38%)	6 (7.89%)	0.79
Hypotension	9 (11.68%)	20 (26.31%)	0.03^#^
Shivering	5 (6.49%)	14 (18.42%)	0.04^#^
Nausea	10 (13.15%)	18 (25%)	0.14
Dyspnoea	0	0	NA
Pruritis	24 (31.16%)	22 (28.94%)	0.80
Postoperative			
Nausea/vomiting	8 (10.38%)	13.15% (n=10)	0.80
PDPH	5 (6.49%)	9.21% (n=7)	0.76
TNS (1st post-op day)	3 (3.89%)	4 (5.26%)	0.87
TNS (7th post-op day)	None	None	NA
Permanent nerve injury	None	None	NA

**Figure 2:**
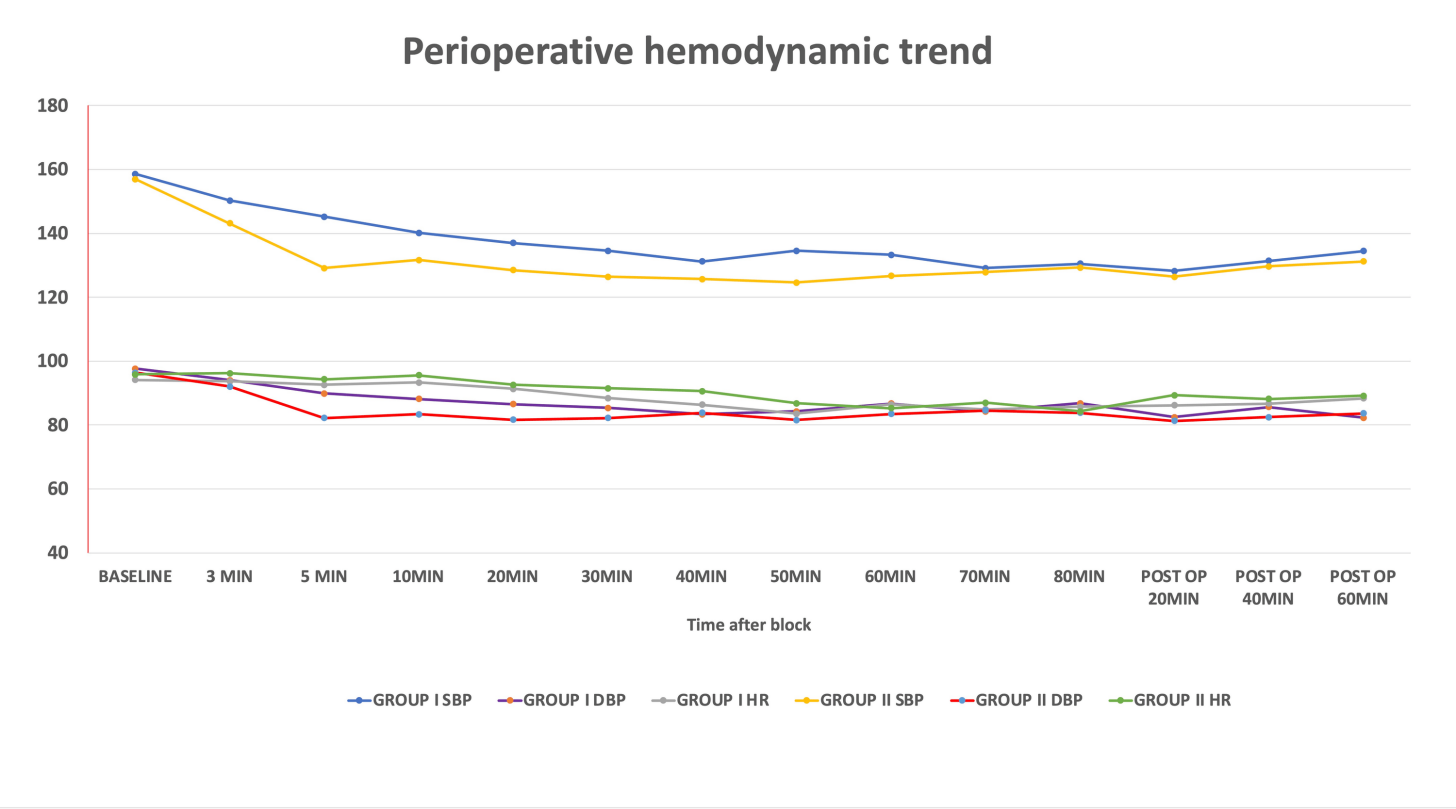
Perioperative hemodynamic trend SBP: systolic blood pressure; DBP: diastolic blood pressure

Similarly, the mean vasopressor (ephedrine) requirement was significantly lower in Group I (8.57 ± 1.32 mg) compared to Group II (14.41 ± 2.62 mg), with a mean difference of 5.84 mg (95% CI: 5.12-6.56; p < 0.001). Episodes of severe hypotension occurred in 2 patients in Group II, while none were reported in Group I. The incidence of bradycardia was slightly higher in Group I (10.38%; n=8) than in Group II (7.89%; n=6), but this difference was not statistically significant (p = 0.794). These trends are shown in Figure 2.

In secondary outcomes, sensory and motor block durations were significantly shorter in Group I (p = 0.001). Group I had a higher number of patients with Bromage Grade 3 block (n = 60) compared to Group II (n = 69), though this was not statistically significant (p = 0.10) (Table 4). The Visual Analog Scale (VAS) score at the end of surgery was comparable between the groups (1.39 ± 0.49 in Group I vs. 1.21 ± 0.61 in Group II; p = 0.065).

**Table 4:** Comparison of sensory and motor block characteristics across groups Data presented as n (%) or Mean ± SD; #: statistically significant; p < 0.05 was considered significant

Parameter	Group I (N = 77)	Group II (N =76)	P-value
Sensory block duration in minutes Mean ± SD	132.61 ± 13.81	148.48 ± 14.52	0.001^#^
Motor block duration in minutes Mean ± SD	102.23 ± 11.13	124.01 ± 11.01	0.001^#^
Intensity of motor block (Modified Bromage Scale)			
Grade 1 (n)	5	1	0.001^#^
Grade 2 (n)	12	7	0.02^#^
Grade 3 (n)	60	69	0.10

The incidence of intraoperative shivering was significantly lower in Group I (6.49%) compared to Group II (18.42%) (p = 0.04). Other adverse effects, including nausea, pruritus, and postoperative complications such as PDPH and transient neurological symptoms (TNS), were comparable between groups (Table 3).

Neonatal outcomes were comparable in both groups. The median (IQR) Apgar score at 1 minute was 6.0 (5.0, 6.0) in Group I and 6.0 (5.0, 7.0) in Group II, with 49.35% and 56.5% of neonates scoring <7, respectively (p = 0.465). At 5 minutes, the median (IQR) Apgar score was 8.0 (7.0, 9.0) in Group I and 8.0 (7.0, 8.0) in Group II (p = 0.436). Intubation was required in 6 neonates in Group I and 5 in Group II. NICU admissions were similar (9 in Group I vs. 11 in Group II). NICU admissions were similar in both groups (9 in Group I vs. 11 in Group II). The most common reason for admission in both groups was low birth weight (5 vs. 5), followed by low Apgar scores at 5 minutes (4 vs. 5). Patient satisfaction, assessed via a 5-point Likert scale at 24 hours, was significantly higher in Group I (50.65% highly satisfied) compared to Group II (38.16%) (p = 0.033). Surgeon satisfaction scores did not differ significantly between the groups (Table 5).

**Table 5:** Comparison of patient and surgeon satisfaction scores across study group Data presented as n; #: statistically significant; p < 0.05 was considered significant

Satisfaction level	Patient satisfaction score			Surgeon satisfaction score		
	Group I (N = 77)	Group II (N = 76)	p-value	Group I (N = 77)	Group II (N = 76)	p-value
Highly satisfied	39	25	0.033^#^	34	29	0.512
Satisfied	25	29	0.689	28	30	0.740
Neutral	9	14	0.356	8	7	0.959
Dissatisfied	4	8	0.229	6	8	0.588
Highly dissatisfied	0	0	1.000	1	2	0.620

## Discussion

Spinal anesthesia is considered the safest approach for cesarean section in patients with preeclampsia; however, managing hypotension remains a significant challenge for anesthesiologists during the procedure [12,13]. The present study offers a direct comparison between thoracic and lumbar spinal anesthesia in preeclamptic patients undergoing cesarean section, emphasizing the impact on hemodynamic stability. A significantly lower incidence of hypotension, reduced vasopressor requirement, and higher patient satisfaction were observed in the thoracic spinal group. These findings support the hypothesis that segmental thoracic spinal anesthesia, with its localized effect and lower anesthetic dose, may offer better hemodynamic control in this high-risk population.

In this study, 1.5 mL of hyperbaric bupivacaine combined with fentanyl was used for thoracic spinal anesthesia. This dose selection aligns with prior studies involving low-dose thoracic segmental blocks. Ellakany administered 2 mL for abdominal cancer surgery, while Chauhan et al. successfully used 1.2 mL in a cesarean section for a preeclamptic woman with pemphigus vulgaris [9,14]. The reduced dose requirement in thoracic spinal anesthesia is attributed to anatomical factors (such as lower CSF volume and thinner thoracic nerve roots), which enhance local anesthetic efficacy and hasten block onset [15,16]. In our study, sensory and motor block durations were significantly shorter in the thoracic group, consistent with the findings of Nagar et al. [17]. Although the postoperative VAS score was slightly higher in the thoracic spinal group, this may be attributed to the use of a lower intrathecal dose and reduced intensity of motor blockade in this group. Despite this trend, the difference was not statistically significant (p = 0.065), and both groups remained well within acceptable pain control levels. However, a dedicated study focusing specifically on postoperative analgesia duration and quality would be valuable to further explore and validate this observation.

The overall incidence of hypotension in our cohort was lower than the 40-55% typically reported in normotensive parturients. This aligns with prior evidence suggesting that preeclamptic patients are relatively protected due to chronic vasoconstriction and elevated endogenous vasopressors [10]. The thoracic approach further reduced hypotension, likely by restricting sympathetic blockade to fewer dermatomes and minimizing systemic vascular resistance (SVR) drop. The preservation of lower limb motor function may have also supported venous return via the calf muscle pump. Similar findings were reported by Ellakany (20% hypotension) and Chandra et al. (18%) with thoracic spinal anesthesia in non-obstetric settings [9,18].

We also observed a lower incidence of intraoperative shivering and nausea in the thoracic spinal group. Though prior studies have not directly compared these effects between thoracic and lumbar approaches, reduced sympathetic and motor blockade in the thoracic group may explain these trends. Our findings are in line with studies reporting 9-20% shivering following intrathecal opioid use [19,20]. The reduced incidence of nausea can be attributed to the lower incidence of hypotension, as previous studies have reported a direct correlation between intraoperative hypotension and nausea [21,22]. The higher patient satisfaction observed in the thoracic spinal group can also be explained by the reduced occurrence of these complications.

Contrary to prior concerns, bradycardia incidence was comparable between groups, likely due to similar block heights and lower anesthetic volume in the thoracic group, limiting cephalad spread. Previous case reports on thoracic spinal anesthesia for cesarean sections noted no significant bradycardia [14,23]. The primary concern with thoracic spinal anesthesia remains the risk of transient or permanent neurological sequelae; however, no long-term complications have been reported in the literature or in this study [24-26].

This study has several limitations. First, it was an investigator-initiated trial conducted at a single center, which may limit the generalizability of the findings. Second, the sample size was modest and calculated based primarily on hemodynamic parameters; hence, rare complications or less frequent outcomes may have been missed. Third, the inclusion of only pre-booked patients may introduce selection bias, as it excludes emergency or unscheduled cases. Lastly, the open-label design without blinding of participants or investigators could have introduced performance or observer bias in subjective outcome assessments, such as VAS scores or satisfaction ratings. Future research should focus on large-scale, multicenter randomized trials involving diverse patient populations to validate and expand upon these findings.

## Conclusions

Thoracic spinal anesthesia is a safe and effective alternative to lumbar spinal anesthesia for cesarean delivery in preeclamptic patients, providing better hemodynamic stability, fewer intraoperative complications, and higher patient satisfaction, without compromising neonatal outcomes. Given its advantages in this high-risk population, it may be preferred where expertise and appropriate patient selection are ensured. For further application and wider clinical adoption, larger multicenter trials are recommended.
